# What Is the Mechanism Driving the Reduction of Cardiovascular Events from Glucagon-like Peptide-1 Receptor Agonists?—A Mini Review

**DOI:** 10.3390/molecules26164822

**Published:** 2021-08-10

**Authors:** Jared Berndt, Soo Liang Ooi, Sok Cheon Pak

**Affiliations:** 1 School of Dentistry and Medical Sciences, Charles Sturt University, Bathurst, NSW 2795, Australia; jared.berndt@lilly.com (J.B.); sooi@csu.edu.au (S.L.O.); 2 Eli Lilly Australia Pty. Ltd., West Ryde, NSW 2114, Australia

**Keywords:** glucagon-like peptide-1, dipeptidyl peptidase-4, haemoglobin A1c, systolic blood pressure, atherosclerosis, mononuclear cells, vascular smooth muscle cells, mitochondria, endothelial cells

## Abstract

Glucagon-like peptide-1 receptor agonists (GLP-1 RAs) are considered the standard of care for type 2 diabetes in many countries worldwide. These molecules have profound anti-hyperglycaemic actions with a favourable safety profile. They are now being considered for their robust cardiovascular (CV) protective qualities in diabetic patients. Most recent CV outcome trials have reported that GLP-1 RAs reduce major adverse cardiovascular events (MACE). Furthermore, the GLP-1 RAs seem to target the atherosclerotic CV disease processes preferentially. GLP-1 RAs also improve a wide range of routinely measured surrogate markers associated with CV risk. However, mediation analysis suggests these modest improvements may contribute indirectly to the overall anti-atherogenic profile of the molecules but fall short in accounting for the significant reduction in MACE. This review explores the body of literature to understand the possible mechanisms that contribute to the CV protective profile of GLP-1 RAs.

## 1. Introduction

Diabetes is a global medical epidemic growing each year; it is also a major risk factor for cardiovascular disease (CVD) and associated morbidity. Furthermore, macrovascular complications account for most hospitalisations and deaths for patients with diabetes [1]. Glucagon-like peptide-1 (GLP-1) receptor agonists (RAs) are considered the standard of care for treating type 2 diabetes (T2D) by the American Diabetes Association [2]. GLP-1 RAs show a robust and sustained reduction in blood glucose markers with a favourable safety profile. In recent years, they are equally considered for their impressive cardiovascular (CV) protection benefit [3].

GLP-1 RAs are synthetic forms of the native GLP-1 produced in the L cells of the small intestine. Native GLP-1 is released from the gut in response to oral glucose intake, and this is commonly referred to as the incretin effect [4]. Endogenous GLP-1 has a plethora of actions throughout the body, including stimulating glucose-mediated insulin release from pancreatic beta cells, inhibiting glucagon secretions from pancreatic alpha cells, slowing gastric emptying, and promoting satiety signals in the central nervous system [5]. Native GLP-1 is rapidly degraded by the enzyme dipeptidyl peptidase-4 (DPP-4) in physiologic conditions. GLP-1 RAs are engineered to be resistant to DPP-4, thus they can augment the incretin effect [6]. GLP-1 receptors (GLP-1Rs) are widely distributed throughout the CV system, and subsequently, GLP-1 RAs have been shown in large cardiovascular outcome trials (CVOTs) to elicit CV protection, irrespective of their glucose-lowering ability [7].

GLP-1 RAs have been prescribed for primary and secondary prevention of CVD [2]. However, the primary mechanism that is driving CV protection remains enigmatic. Table 1 shows a list of CVOTs reporting the MACE reduction effects of different GLP-1 RA molecules compared to the placebo. A recent meta-analysis confirms that the overall class of GLP-1 RAs significantly reduced major adverse cardiovascular events (MACE) by 12% (Hazard ratio = 0.88, 95% confidence interval: 0.84–0.94; *p* < 0.001) [3]. Efpeglenatide was not included in this meta-analysis as its results were published later. As shown in Table 1, five out of seven CVOTs reported significant reductions of MACE versus placebo, whereas two of the studies reported neutral results. Interestingly, both GLP-1 RAs that produced a neutral effect had a daily dosing regimen instead of weekly [8,9].

Another GLP-1 RA, taspoglutide, is not mentioned in Table 1 as it was never commercialised due to tolerability issues that significantly disrupted the associated CVOT. Comparable data are thus unavailable [14]. Nevertheless, a meta-analysis combining all available randomised control trials on taspoglutide has reported its CV safety [15]. One more incretin mimetic of interest is tirzepatide. This novel dual GLP-1Rs/glucose-dependent insulinotropic polypeptide co-agonist has the most promising effect on weight loss and haemoglobin A1c (HbA1c) to date [16]. Tirzepatide is currently being investigated in a large CVOT, with results expected in the coming years [17].

The MACE components that show the most prominent trend in reduction appear to be associated with atherosclerotic pathways [3]. The finding suggests that mechanistic analysis may detect anti-atherogenic properties of GLP-1 RAs throughout the vascular tissue. Additionally, GLP-1Rs are ubiquitously expressed throughout the cells that are involved in atherosclerosis pathogenesis [18]. The results of these CVOTs pose further research questions as to what predominant mechanism is driving the CV protection from these molecules. The CV surrogate markers measured in these studies are not able to fully account for the extensive CV benefit produced; hence, there is an identifiable gap in the literature. Therefore, there is a need to understand the cause and effect of GLP-1 RAs on CV benefit within the relevant CV tissues.

The interaction of GLP-1 RAs with the GLP-1Rs and the subsequent intracellular pathways are well defined in tissues outside the CV system [19]. Previous reviews have looked at both endogenous and exogenous incretins [6] and DPP-4 inhibitors [20] in the context of CVD. Additionally, a more recent review has a similar research question and includes a comparable array of GLP-1 RAs. However, it has a wider focus on both diabetic kidney disease and CVD [21]. This mini-review differs from the previous research in that it is addressing exclusively injectable synthetic GLP-1 RAs’ effect on CVD as opposed to endogenous GLP-1. In addition, this paper focuses primarily on atherosclerotic CVD consistent with the data in CVOTs.

**Figure 1:**
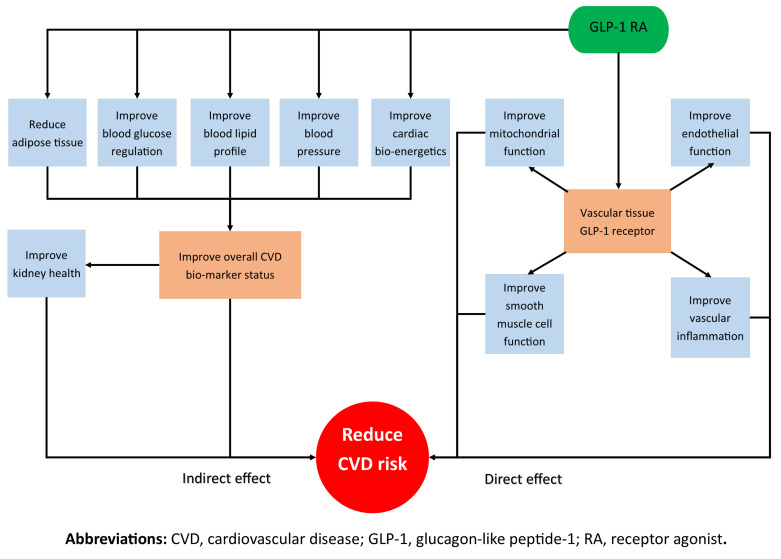
A schematic that outlines the direct and indirect actions of GLP-1 RAs in reducing CVD risk.

As shown in Figure 1, this review will first look at the possible indirect effects of the GLP-1 RAs on CVD throughout the entire body. The review will also analyse the effect of incretin mimetics on well-understood CVD surrogate markers, including blood glucose, blood pressure (BP), lipids and weight. The paper will then explore the direct relationship between GLP-1 RAs and atherosclerosis pathogenesis by systematically separating the pre-clinical studies into structural components of the vasculature to help the readers conceptualise how the molecules affect different tissues and interact with different cells.

## 2. Indirect Effects of GLP-1 RAs on CVD Risk Reduction

### 2.1. Kidneys

Kidney outcomes in all the included GLP-1 RA CVOTs are secondary endpoints and therefore are exploratory without the statistical power to claim any specific renal benefit. A pooled analysis across all the CVOTs found that the composite kidney outcome reduced by 17% compared to the placebo, and the largest data signal was for a reduction in albuminuria [22]. However, the variances of reno-protection between molecules could be attributed to differences in trial design [23]. In a recent review, van Baar et al. [24] suggest that GLP-1 RAs can protect kidney health directly or indirectly. GLP-1 RAs have been shown to reduce blood glucose and modestly improve BP, adiposity and serum lipids, which, when aggregated, can indirectly affect renal health. Moreover, the kidneys widely express the GLP-1Rs, although their exact location within the tissue remains unknown [25].

A recent review summarises the body of literature describing primarily natriuretic and an immunomodulatory effect of GLP-1 RAs, causing the reno-protective effect [26]. The consistent data trends seen across the body of literature has validated the need for further analysis. Currently, there is an ongoing clinical trial with semgalutide exploring its effect in patients with chronic kidney disease. This study will be the first large scale GLP-1 RA study with kidney outcomes forming the primary endpoint [27].

### 2.2. Blood Glucose, BP, Lipids and Weight

GLP-1 RAs significantly reduce HbA1c compared to the placebo, and as reported in a mediation analysis of dulaglutide, HbA1c only mediates a modest proportion of the overall CV benefit [3,28]. Glucose-lowering does decrease CV risk; however, the method of lowering blood glucose matters. GLP-1 RAs lower the blood glucose without additional CVD burden. In contrast, other anti-hyperglycaemic molecules may counteract the CV protective effect of glucose-lowering through harmful off-target mechanisms [29,30]. Out of the CVOTs, semaglutide has shown the most significant ability to reduce weight [10]. Additionally, weight loss driven by GLP-1 RAs is likely to influence the overall CV protective profile of the molecules positively [10,31,32]. However, most molecules available fall short of clinically significant weight loss that would sufficiently account for the overall CV protection [33]. On the contrary, subgroup analysis of dulaglutide by Gerstein et al. [7] has shown that the CV benefit from GLP-1 RAs occurs irrespective of weight loss.

The CVOTs report modest improvement in the lipid profile from GLP-1 RAs treatment. However, there was a significant proportion of patients in these studies receiving lipid modulating therapy [3,7,22]. A recent systemic review reported that GLP-1 RAs can modestly alter postprandial serum lipids via the alteration of hepatic triglyceride output [34]. Furthermore, the review proposed additional mechanisms that improve lipid metabolisms, such as improved peripheral bio-energetics and increased endogenous insulin functionality [34]. Despite most patients in the large CVOTs being on the standard hypertension care, GLP-1 RAs can modestly augment systolic BP reduction [3]. The mechanism has been investigated in vitro whereby liraglutide caused an increase in vasodilation in the arterial vasculature and decreased vascular contraction, causing a reduction in systolic BP [35]. Notwithstanding, a mediation analysis of dulaglutide reports that BP reduction does not significantly contribute to the overall CV protection [28].

### 2.3. Myocardium

The body of literature suggests that GLP-1 RAs directly affect the myocardial tissue and indirectly change the peripheral circulating energy substrates [36]. A recent systematic review cited limited expression of GLP-1Rs in the ventricular myocardium, suggesting the increase in cardiac efficiency must be a consequence of GLP-1Rs stimulation in the coronary vascular smooth muscle cells [37]. Moreover, the remainder of the protective benefit must be derived from the peripheral vasculature with a high expression of GLP-1Rs [37]. Another possible mechanism in which GLP-1 RAs can influence the myocardial tissue is through bioenergetics. The myocardium has metabolic flexibility using glucose and fat substrates for adenosine triphosphate production in a healthy person. In the context of T2D, there is a marked increase in circulating fatty acids. Subsequently, the predominant substrate for myocardial cells becomes fatty acids instead of having a mixture of fuels [36].

Improved blood glucose control may also enhance the availability to use alternate substrates, such as ketones and lactate, for fuel. This ’thrifty substrate’ hypothesis has been proposed for the sodium-glucose cotransporter 2 inhibitors, another T2D medication class with a proven CV benefit [38]. Incretin molecules have been shown to potentially reduce the severity of myocardial infarction (MI) by a reduction of infarct size, as reported in pre-clinical studies [39]. Incidentally, GLP-1 RAs have a structural resistance to DPP-4-mediated degradation and do not form the same metabolites as endogenous GLP-1. These endogenous metabolites may have their own unique CV protective mechanisms outside of GLP-1R stimulation [37]. Moreover, a recent review suggests that DPP-4 inhibition may be associated with several anti-atherogenic pathways independent of an increase in endogenous GLP-1 and glucose-dependent insulinotropic polypeptide [20].

## 3. Direct Effects of GLP-1 RAs on CVD Risk Reduction

### 3.1. Immune Response

Atherosclerosis is a condition characterised by chronic inflammation in the vascular walls. The dominant immune cells implicated in the formation of atherosclerotic plaques are mononuclear leukocytes. GLP-1 RAs may contribute to modulating the immune response to atherogenic environments, which can, in turn, assist in the stabilisation of existing lesions. Lixisenatide is reported to bind to monocytes’ GLP-1Rs and modulate the immune response via increased intracellular 5${}^{\prime }$-AMP-activated protein kinase (AMPK) [40]. Similar results have been replicated with liraglutide downregulating proinflammatory cytokines via an increase in AMPK [41].

Additionally, through analysing messenger ribonucleic acid expression in GLP-1 RA-treated cells, there are reportedly decreased levels of proinflammatory mediators and their precursors of tumour necrosis factor (TNF)-α, monocyte chemoattractant protein-1 and nuclear factor (NF)-κB [42]. Moreover, when genetically prone atherosclerotic mice were treated with semaglutide or liraglutide, they had reduced plaque formation as well as attenuated levels of TNF-α and interferon-γ [43]. Similar results were replicated in a murine model where liraglutide was able to reduce the total plaque burden and increase the stability of existing plaques. Interestingly, when co-administering a GLP-1R antagonist, the effect of liraglutide on plaques was able to be partly attenuated [44]. This finding suggests that GLP-1 RAs may have immunomodulating effects in the vascular walls and may prevent the development of atherosclerotic plaques in patients that express high CV risk phenotypes. These results also indicate that the effects of GLP-1 RAs are likely to be driven significantly from GLP-1R activation.

Pro-atherogenic inflammation causes the recruitment of monocytes into the vascular wall. The subsequent transformation of monocytes into macrophages and eventually into foam cells is central to the pathogenesis of atherosclerosis [45]. Cultured monocytes treated with exenatide show an increase in autophagy of chemotactically recruited monocytes [46]. Macrophages seem to have different phenotypes that drive different behaviours. Exenatide has been reported to favourably manipulate the phenotype of the macrophages from M1 to M2 [47].

Furthermore, liraglutide also shifts the proportion of M2 macrophages compared to M1 in mice treated with placebo [48,49]. These changes to the macrophage phenotype reduce deleterious changes in the vascular wall and promote the favourable stabilisation of atherosclerotic lesions. Liraglutide-treated mice were able to recruit a more favourable immune infiltrate into atherosclerotic lesions, which reduces overall atherosclerotic burden [48,49]. Furthermore, exenatide can attenuate reactive oxygen species (ROS) production from stressed macrophages [50]. Such observation further suggests the ability of GLP-1 RAs in modulating harmful immune responses that drive the formation of plaques.

### 3.2. Vascular Smooth Muscle Cells (VSMC)

VSMC dysfunction can drive pro-atherogenic conditions in the vascular walls, including disrupting vascular tone and increasing the switch from a contractile to a proliferative phenotype. This phenotype change in the VSMC may drive pathogenic remodelling in the context of atherogenic signals [51]. Exenatide has demonstrated the ability to mitigate this dysfunction by modulating the phenotypic switching of VSMC in cell culture [52]. The authors reported that the stimulation of GLP-1R demonstrated an increase in AMPK phosphorylation and an increase in intracellular Sirtuin1 and Forkhead box O3. Therefore, these pathways are the likely mediators between GLP-1 RAs and the correction of the VSMC function [52].

In an atherosclerotic murine model, liraglutide was able to mitigate the actions of angiotensin II (ANG II) in VSMC, which delayed the progression of plaque formation and promoted the stabilisation of existing lesions [53]. The ability of GLP-1 RAs to increase intracellular AMPK and subsequent downstream consequences on cell cycling may be responsible for the attenuation of pathological remodelling of the vasculature [53]. In addition, cultured aortic VSMC treated with exenatide ameliorate the deleterious effects of ANG II by offsetting extracellular signal-regulated kinase (ERK) and c-Jun N-terminal kinase phosphorylation [54]. These pathways are typically stimulated by ANG II and cause pro-atherogenic remodelling. The ability to mitigate atherogenic signals by GLP-1 RAs may also be through the inhibition of the phosphoinositide 3-kinases and protein kinase B pathways [55].

A review explores the important roles of matrix metalloproteinases (MMPs) and tissue inhibitors of metalloproteinases (TIMPs) in atherosclerosis development [56]. Recent pre-clinical data suggest that GLP-1 RAs may lead to CV protection through the modulation of the ratio of MMPs and TIMPs. In human endothelial cells, exenatide caused suppressed levels of MMPs and an increase in TIMPs [57]. In another in vitro study, exenatide has also been reported to favourably modulate the expression of MMPs and TIMPs in coronary artery smooth muscle cells [58]. The inhibition of the NF-κB and Akt signalling pathway has been identified as the likely mediator between exenatide and MMPs in the two studies mentioned above, respectively [57,58].

Interestingly, a plausible mechanism of NF-κB inhibition may be through the GLP-1 RA stimulation of adipose adiponectin (APN) [59]. Exenatide has been reported to upregulate APN expression in a murine model [60]. Platelet-derived growth factor (PDGF) is an additional candidate for the underlying mechanism of GLP-1 RAs to modulate the development of atherosclerotic plaque. It has been reported that several GLP-1 RAs are able to mitigate PDGF-induced proliferation of VSMC, influencing the local atherosclerosis physiology [37].

### 3.3. Mitochondria

Another possible protective mechanism of GLP-1 RAs against CVD may be through actions in the mitochondria. Liraglutide-treated rats exposed to a known stressor were able to mitigate mitochondrial dysfunction in VSMC [61]. In contrast, their counterparts treated with a GLP-1R antagonist demonstrated an increase in mitochondrial dysfunction. The authors concluded that liraglutide triggers an increase in CV protection via direct binding to VSMC GLP-1Rs and upregulating various intracellular pathways involved in mitochondrial health [61].

GLP-1 RAs may be able to decrease mitochondria-driven apoptosis in CV tissue. Cells treated with exenatide consistently showed decreased caspase-3 activity, a known marker of apoptosis [62,63]. In another study, exenatide demonstrated the ability to mitigate the effects of TNF-α in treated cells by attenuating excessive ROS production and upregulating various other pathways that are pertinent for mitochondrial integrity [64]. Moreover, an in vitro model of murine cardiomyocytes stressed by hypoxic conditions reported decreased mitochondrial ROS production in exenatide-treated cells [62]. Additionally, several intracellular signals associated with mitochondrial health and function were upregulated in those cells.

When exenatide was administered to MI-prone mice, it improved CV performance and decreased deleterious remodelling. Furthermore, exenatide also attenuated attenuated ROS production and improved mitochondrial integrity in stressed cardiomyoblasts [63]. A potential mechanism in which GLP-1 RAs can attenuate ROS production in the mitochondria could be the lectin-like oxidised low-density lipoprotein receptor-1 (LOX-1). Liraglutide could function as a mediator between LOX-1 and mitochondrial ROS production, as demonstrated in a recent study. The researchers were able to blunt the protective effects of liraglutide on ROS production by manipulating LOX-1 activity through antibody treatment [65].

### 3.4. Endothelium

GLP-1 RAs may buffer the vascular endothelial cells from deleterious signalling exhibited by oxidised low-density lipoprotein (ox-LDL). In vitro cell culture of human aortic cells infused with ox-LDL and dulaglutide showed reduced inflammatory cell adhesion, a known precursor of atherosclerosis formation. The biochemical analysis of cell signalling proteins suggests that dulaglutide may buffer the effects of ox-LDL on Krüppel-like factor 2 (KLF2) transcription factors [66]. The KLF2 pathway is pertinent to the maintenance of vascular integrity, and GLP-1 RAs attenuate the effect of ox-LDL on this pathway. The same effect was observed with liraglutide in another study, which offered further insights into the intracellular signalling pathway [67]. The authors concluded that GLP-1 RA signalling was dependent on ERK 5, as benefits from liraglutide were mitigated with the inhibition of the ERK 5 pathway [67].

GLP-1 RAs have been reported to decrease vascular adhesion molecules in an in vitro model. Mice treated with liraglutide produced correspondingly improved endothelial function [68]. Additionally, liraglutide was able to attenuate the elevation of vascular cell adhesion molecule-1, intercellular adhesion molecule-1 and E-selectin in liraglutide-treated endothelial cells, likely via the activation of calcium/calmodulin-dependent protein kinase I and AMPK. Interestingly, the co-administration of a GLP-1R antagonist attenuated the effect of liraglutide [68]. The finding suggests that the effect of GLP-1 RAs on the endothelium is mediated through GLP-1R activation.

Endothelin-1 (ET-1) is a potent vasoconstrictor, and its upregulation is typically evident in the endothelium of atherosclerotic disease. As such, elevated ET-1 is implicated in a wide range of CVD pathologies. When cells were treated with liraglutide, reduced ET-1 activity was observed, possibly due to the decreased NF-κB activity [69]. The ability of GLP-1 RAs to inhibit NF-κB is consistently reported in the body of literature.

## 4. Conclusions

Meta-analysis of the GLP-1 RA CVOTs suggests a class effect of CV benefit. However, there were reported differences among the molecules with regards to MACE reduction. Some of the inconsistency may be attributed to differences in study design. Incidentally, the two molecules that did not meet their primary endpoint involved daily dosing instead of weekly. Moreover, the reported pre-clinical trials did not represent a balanced proportion of all the GLP-1 RA molecules; therefore, any differences in their molecular structures that may influence the CV benefits in pre-clinical trials are difficult to confirm.

Additionally, the CVOTs used GLP-1 RAs on top of the standard of care; therefore, it must be considered that these molecules may be working synergistically with another CVD pharmacotherapy. Moreover, the study population in these clinical trials was also diabetic. The CV benefit is yet to be demonstrated in patients without T2D. Furthermore, there seem to be differences among GLP-1 RAs in their ability to reduce various surrogate markers of CVD; however, all molecules demonstrated an overall favourable profile compared to the placebo. There are distinct differences in the metabolism of GLP-1 RAs and native GLP-1, both potentially offering unique and overlapping CV benefits.

GLP-1 RAs seem to elicit CV protection both directly in the vasculature and indirectly in the periphery. However, what remains unclear is the proportion of this CV protective mechanism that is driven from local GLP-1R stimulation versus the cumulative peripheral effects that may indirectly improve vasculature function. Moreover, several in vitro studies using GLP-1R antagonists as a control suggest that a significant proportion of the effects of GLP-1 RA is a direct consequence of GLP-1R stimulation and subsequent intracellular cascades. Further investigation of the specific intracellular pathways stimulated by GLP-1R activation is needed.

The reported mechanistic studies consistently demonstrated the attenuation of atherosclerosis-associated processes and seemed to promote signals consistent with the stabilisation of existing lesions. Further investigation is needed to properly quantify the proportional influence of suggested mechanisms of GLP-1 RA CV protection. In conclusion, GLP-1 RAs likely have pleiotropic anti-atherogenic effects throughout the CV system and lead to other favourable metabolic changes in the periphery to produce the overall CV benefit.

## Figures and Tables

**Table 1:** The effects of GLP-1 RAs on relative MACE reduction compared to placebo: The results from CVOTs.

Author (Year)	Molecule	N	Hazard Ratio (95% CI)	*p*-Value
Pfeffer et al. (2015) [8]	Lixisenatide	6068	1.02 (0.89–1.17)	0.81
Marso et al. (2016) [10]	Semaglutide	3297	0.74 (0.58–0.95)	0.02 *
Holman et al. (2017) [9]	Exenatide	14,752	0.91 (0.83–1.00)	0.06
Hernandez et al. (2018) [11]	Albiglutide	9463	0.78 (0.68–0.90)	0.0006 *
Zinman et al. (2018) [12]	Liraglutide	9340	0.87 (0.78–0.97)	0.01 *
Gerstein et al. (2019) [7]	Dulaglutide	9901	0.88 (0.79–0.99)	0.026 *
Gerstein et al. (2021) [13]	Efpeglenatide	4076	0.73 (0.58–0.92)	0.007 *

Abbreviation: CI, confidence interval; CVOTs, cardiovascular outcome trials; MACE, major adverse cardiovascular events; N, number of participants; * Significant results with *p* < 0.05.

## Abbreviations

The following abbreviations are used in this manuscript:

AMPK	5′-AMP-activated protein kinase
ANG II	Angiotensin II
APN	adipose adiponectin
BP	Blood pressure
CVOTs	Cardiovascular outcome trials
CV	Cardiovascular
CVD	Cardiovascular disease
DPP-4	Dipeptidyl peptidase-4
ERK	Extracellular signal-regulated kinase
ET-1	Endothelin-1
GLP-1	Glucagon-like peptide-1
GLP-1R	Glucagon-like peptide-1 receptor
KLF2	Krüppel-like factor 2
LOX-1	Lectin-like oxidised low-density lipoprotein receptor-1
MACE	Major adverse cardiovascular events
MI	Myocardial infarction
MMPs	Matrix metalloproteinases
NF	Nuclear factor
ox-LDL	Oxidised low-density lipoprotein
PDGF	Platelet-derived growth factor
RAs	Receptor agonists
ROS	Reactive oxygen species
T2D	Type 2 diabetes
TIMPs	Tissue inhibitors of metalloproteinases
TNF	Tumour necrosis factor
VSMC	Vascular smooth muscle cells
